# Medicines and the media: news reports of medicines recommended for government reimbursement in Australia

**DOI:** 10.1186/1471-2458-13-489

**Published:** 2013-05-21

**Authors:** Jane Robertson, Emily J Walkom, Marc D Bevan, David A Newby

**Affiliations:** 1 Clinical Pharmacology, School of Medicine and Public Health, The University of Newcastle, Newcastle, NSW, Australia

**Keywords:** Pharmaceuticals, PBAC, Media, Australia

## Abstract

**Background:**

Previous analyses of the listings of trastuzumab on the Australian Pharmaceutical Benefits Scheme (PBS) and HPV vaccine on the National Immunisation Program (NIP) suggest a media influence on policy makers. We examined the timing and content of Australian newspaper reports of medicines in relation to Pharmaceutical Benefits Advisory Committee (PBAC) decisions.

**Methods:**

We identified newspaper reports (2005-2008) of medicines recommended for PBS listing in 2006–2007, analysing the content for mentions of the medicine, PBS and medicine costs to the patient and the government and counting the numbers of articles published in the six months before, the month of, and the six months after the relevant PBAC meeting. Case studies examined reporting for infliximab for Crohn’s Disease, pemetrexed for mesothelioma, and ADHD (Attention Deficit Hyperactivity Disorder) medicines atomoxetine and methylphenidate.

**Results:**

Of 79 eligible medicines, 62 had news reports. Most often reported were HPV vaccine (1230 stories), trastuzumab (410), pemetrexed (83), botulinum toxin (71), lapatinib (65), methylphenidate (57), atomoxetine (54), infliximab (49), rotavirus vaccine (45). Eighteen medicines had ≥20 news reports (total 2350 stories); nine of these cost more than AU$10,000 per course or year of treatment. For these 18 medicines, 31% of stories appeared in the six months prior to the PBAC meeting, 14% in the meeting month and 33% in the six months post-meeting. 38% of the stories had ≥3 medicine mentions, 37% referred to the PBS, 24% to cost to the patient, and 9% cost to Government.

There was active patient lobby group campaigning in support of listing of infliximab and pemetrexed; the stories for ADHD were often more negative, referring to the dangers of the medicines and sometimes questioning the appropriateness of treatment and public subsidy. There was little discussion of the PBAC’s evidence-based decision-making processes.

**Conclusions:**

While there was no general trend to increased news reporting associated with PBAC meetings, some drugs did attract media attention. With more new and expensive drugs, decisions on public funding will become increasingly difficult. The media have an important role in enhancing public understanding of the issues around resource allocation. Specialist journalists, guidelines and checklists may help reporting.

## Background

The news media are an important source of information on health and medical advances for consumers. However, the quality of media coverage is variable, with concerns about exaggerated claims of benefits, inadequate coverage of harms, a lack of information on treatment costs, and limited use of independent expert opinion to interpret the balance of benefits and harms for consumers [1-4]. Reporting about medicines varies in its focus and impacts. Newspaper stories around the time of marketing of a new medicine can increase public awareness, [5] and may promote patient requests for the product [6,7]. Business news reports relate to share prices and projected profits for pharmaceutical companies; while adverse publicity on drug safety can have dramatic negative effects on market values [8,9].

Australia’s Pharmaceutical Benefits Scheme (PBS) is a publicly funded insurance scheme aimed at providing universal affordable access to prescription medicines [10]. Approximately 80% of the prescription medicines used in Australia are subsidised under the scheme [11]. The private prescription market for medicines not listed on the PBS is estimated to be around 5% of total prescriptions, the remainder funded by other Commonwealth and State programs [12]. All patients contribute a per prescription co-payment until reaching a threshold after which a ‘safety net’ comes into effect; the safety net serves to protect individuals and families with high medication needs [13]. The Pharmaceutical Benefits Advisory Committee (PBAC) is an independent statutory body that makes recommendations to the Minister for Health on the drugs and medicinal preparations to be subsidised on the PBS or included in the National Immunisation Program (NIP). In doing so, the PBAC considers the safety, efficacy and cost-effectiveness of the proposed medicine [10]. Where the PBAC has rejected the application, the sponsor may re-submit any number of times, generally with revised clinical data or clinical claims, changed utilisation estimates or alternate pricing proposals.

Two recent Australian case studies (trastuzumab for breast cancer, Herceptin®) and HPV cervical cancer vaccine, Gardasil®) suggest a media role in increasing political pressure to influence decision-makers [14,15]. The pressure was directed at the Federal Government to commit substantial public money to subsidise an otherwise expensive and mostly unaffordable drug (trastuzumab), and to include the new HPV vaccine in the NIP.

After three rejections by the PBAC for listing for late stage cancer during the 1990s, intensive lobbying before the 2001 Federal election resulted in public subsidisation of trastuzumab in a special program independent of the PBS [14]. In 2006, a “crescendo of media demands” for trastuzumab to be available for early stage cancer preceded PBS listing. Reviewing 43 television news and current affairs stories regarding trastuzumab, MacKenzie *et al.* concluded “*the dominant discourse across the news coverage was that Herceptin was a ‘wonder drug’ made unaffordable to the majority of women with HER2 breast cancer by government indifference, labyrinthine bureaucracy and unacceptable, cruel financial parsimony”*[14]. The initial decision to reject funding of the HPV vaccine led to over 300 newspaper articles and calls by consumers, health professionals and politicians to intervene in the decision-making process [15].

Given these and other examples of media influence on the formulation of health policy, [5,16] we examined the numbers of stories published in Australian newspapers on medicines seeking public subsidy and their timing in relation to PBAC decision-making. We expected there would be increased levels of media reporting in the months leading up to PBAC meetings, these would be highest in the month of the PBAC meeting, and there would be limited media coverage after the positive recommendation to list the medicine on the PBS. In addition, we sought to characterise the reports in terms of mentions of the PBAC and its decision-making, drug costs and budgetary impact of the listing decision.

## Methods

We identified medicines considered by the PBAC for PBS listing in 2006 and 2007 [17]. We searched the ANZ Proquest Newsstand database [18] from 1 July 2005 to 30 June 2008 to find Australian newspaper articles pertaining to these medicines. At the time of this study, the database included more than 40 newspapers, covering the major daily and weekend newspapers of all capital cities and major regional centres [18]. Using generic and trade names, we searched the database for mentions of the medicine in the headline or text of reports and retrieved the full text of each identified article.

We counted the numbers of stories appearing in the six months before, the month of the meeting and six months after the PBAC meeting. Each story was coded for the number of times the medicine name was reported (1, 2 or ≥3 mentions), assuming that more mentions would reflect a greater medicine focus in the newspaper report. Stories identified in the three year time window (2005–2008) were coded for reference to the PBS (either use of the term PBS or reference to Government subsidy or medicine subsidisation), and medicine costs (either patient costs if it was not subsidised, or Government costs for providing subsidised access).

Each story was coded by two raters, with differences in categorisations resolved by consensus or by adjudication using a third reviewer where agreement could not be reached. Descriptive statistics are used to summarise the data.

In addition to the quantitative analysis of the news reports, three case studies where there were larger numbers of newspaper articles published around the time of the PBAC recommendation are used to illustrate the content and tenor of some reporting.

## Results

### Medicines recommended for PBS listing

Seventy-nine medicines receiving a positive PBAC recommendation for listing in 2006 (42 medicines) and 2007 (37 medicines) were identified. Ten of these medicines had more than one application to the PBAC for use in different clinical conditions. Eight medicines were the subject of 50 or more news reports in the eligible time frame (2005–2008), 10 had 20–49 reports, 18 had 10–19 reports, 26 had <10 reports and 17 medicines were not mentioned in any news report (see Appendix I). In total, 2664 stories relating to 62 medicines were identified.

Medicines receiving greatest newspaper coverage were HPV vaccine (1230 stories), trastuzumab (410), pemetrexed (83), botulinum toxin (71), lapatinib (65), methylphenidate (57), atomoxetine (54), Tdap (tetanus, diphtheria and acellular pertussis) vaccine (53), infliximab (49), and rotavirus vaccine (45). The 18 medicines with 20 or more newspaper reports were mentioned in 2350 stories (88% of eligible articles). Nine of these 18 medicines cost more than AU$10,000 per course or year of treatment (see Table 1).

**Table 1:** Medicines with >20 newspaper articles (July 2005-June 2008)

**Generic name, brand name (Indication for PBS listing)**	**No. of articles**	**Number of articles located**		
		**Articles in 6 months prior**	**Articles in PBAC meeting month**	**Articles 6 months post**
HPV vaccine	1230	484	215	409
(NIP)				
Trastuzumab ^†^	410	113	32	104
(Breast cancer)				
Pemetrexed ^†^	83	20	39	16
(Mesothelioma)				
Botulinum toxin	71	11	1	26
(Spasticity)				
Lapatinib ^†^	65	14	4	21
(Breast cancer)				
Methylphenidate	57	12	3	27
(ADHD)				
Atomoxetine	54	6	4	25
(ADHD)				
Tdap vaccine	53	9	4	20
(NIP)				
Infliximab ^†^	49	12	3	32
(Crohn disease, psoriatic arthritis)				
Rotavirus vaccine	45	13	5	16
(NIP)				
Varenicline	39	1	1	24
(Smoking cessation)				
Docetaxel ^†^	36	3	4	17
(Breast cancer, prostate cancer)				
Fentanyl	30	6	4	8
(Severe pain)				
Sildenafil ^†^	28	5	0	0
(Pulmonary hypertension)				
Paclitaxel	27	3	3	11
(Breast cancer)				
Imatinib ^†^	26	2	0	6
(Acute lymphoblastic leukaemia)				
Ranibizumab ^†^	26	4	2	9
(Macular degeneration)				
Natalizumab ^†^	21	2	0	
(Multiple sclerosis)				
**Total**	2350	720	324	783

Abbreviations: *NIP*, National immunisation program; *ADHD*, Attention-deficient hyperactivity disorder; *HPV*, Human papillomavirus; *Tdap*, Tetanus, diphtheria, acellular pertussis.

^†^ Cost more than AUD$10,000 per year or course of treatment.

Of the 18 highly reported medicines, six (33%) were treatments for cancer or other malignancies (docetaxel, imatinib, lapatinib, paclitaxel, pemetrexed, trastuzumab), three were vaccines (HPV, rotavirus, Tdap), two were used to manage Attention Deficit Hyperactivity Disorder (ADHD; methylphenidate, atomoxetine), and five were for severe, often debilitating chronic conditions (infliximab for Crohn’s disease and psoriatic arthritis, fentanyl for severe chronic pain, natalizumab for multiple sclerosis, ranibizumab for macular degeneration, sildenafil for pulmonary hypertension). Botulinum toxin reports in the eligible time period related almost exclusively to its cosmetic ‘Botox’ uses rather than its PBS listing for spasticity. Five of these 18 medicines (infliximab, docetaxel, fentanyl, methylphenidate, varenicline) were the subject of more than one PBS recommendation during the eligible study period increasing the opportunities for news reports for these drugs.

Timing of these 2350 news reports in relation to PBAC meetings is shown in Table 1. Across these 18 medicines, 31% of the reports appeared in the 6 months prior to the PBAC meeting, 14% in the month of the meeting and 33% in the 6 months after the positive PBAC recommendation. For this same subset of 18 medicines, there were ≥3 mentions of the medicine in 38% of the stories, reference to the PBS in 37%, personal cost to the patient of the medicine in 24%, and overall cost to Government in 9% (Table 2). Personal cost in the absence of subsidy was mentioned most often for oncology medicines (docetaxel, lapatinib, pemetrexed, trastuzumab, 51% of stories for these medicines) and the monoclonal antibodies (infliximab, natalizumab, ranibizumab, trastuzumab, 52% of stories). Government subsidy (67.7%) and personal cost of the medicine (48.3%) were mentioned more frequently in the stories for the nine medicines costing more than AU$10,000 per course or year of treatment than overall. However, other high-cost medicines including bortezomib (7 stories) and dasatinib (1 story) received minimal media attention.

**Table 2:** Content of newspaper articles

**Medicine or vaccine (total number of articles)**	**% of articles mentioning **^**#**^			
	**3+ drug mentions**	**Govt subsidy**	**Personal cost**	**Govt cost**
HPV vaccine (n = 1230)	43	20	13	10
Trastuzumab ^†^ (n = 410)	41	67	51	10
Pemetrexed ^†^ (n = 83)	20	99	45	7
Botulinum toxin (n = 71)	8	4	17	1
Lapatinib ^†^ (n = 65)	32	55	42	2
Methylphenidate (n = 57)	70	47	26	18
Atomoxetine (n = 54)	30	67	15	26
Tdap vaccine (n = 53)	9	13	0	0
Infliximab ^†^ (n = 49)	27	82	45	2
Rotavirus vaccine (n = 45)	13	73	24	20
Varenicline (n = 39)	51	51	5	3
Docetaxel ^†^ (n = 36)	31	97	78	19
Fentanyl (n = 30)	13	7	0	3
Sildenafil ^†^ (n = 28)	11	0	0	0
Paclitaxel ^†^ (n = 27)	4	7	0	4
Imatinib ^†^ (n = 26)	54	19	12	0
Ranibizumab ^†^ (n = 26)	42	62	69	19
Natalizumab ^†^ (n = 21)	38	76	62	19
**Total**	38	37	24	9

^†^ Cost more than AUD$10,000 per year or course of treatment.

^#^ Totals do not sum to 100% as an article may be included in more than one category.

While there was no general trend to increased news reporting associated with PBAC meetings, some drugs did attract substantial media attention. We conducted additional qualitative analyses of reports for two medicines (infliximab for the treatment of Crohn’s disease and pemetrexed for mesothelioma) and one class of medicines (ADHD medicines, atomoxetine and methylphenidate).

### Case study – infliximab (5th application for PBAC listing)

There was a peak of reports in the month prior to the positive PBAC recommendation for subsidised use of infliximab in adult Crohn’s disease. In these articles, the case for infliximab was sometimes personalised with identifiable victims *No joy for Sharron after $3000 magic pill rejected*, [19]*Jill Knox may have to remortgage her home*, [20]*Nicole O’Malley’s years of agony*[21]. Patients were described as *let down by the health system*[20]*and suffering needlessly*[22]. Doctors were *forced to offer second-best treatment to desperate people who deserve better*[23].

There was active lobbying by the Australian Crohn’s and Colitis Association [24] specialist physicians and patients [25] (http://www.infliximab.org*)* and a petition of 25,000 signatures was delivered to Health Minister Tony Abbott demanding a listing of the drug [26].

One newspaper view before listing was *that is unacceptable. Actually it is un-Australian. That’s not how we do things*… *a public campaign is the only chance*[27]. After the positive recommendation, newspaper reports noted it was *long overdue*, [28] there was *hip pocket relief*, [29] and that it was c*lose to winning a seven year battle*[30]. The decision was described as a *backflip following extensive lobbying*, [30] commenting *they’ve obviously listened to the public*[31].

### Case study – pemetrexed (4th application for listing)

The story of pemetrexed (marketed as Alimta® in Australia) involved a very public face of a disease resulting from exposure to asbestos. Bernie Banton led the fight on behalf of workers and unions against James Hardie Industries for the establishment of an adequate compensation fund for victims of mesothelioma, only to find himself a victim of the disease.

Before the PBAC recommendation for listing (November 2007), patients were described as innocent victims: *my only crime was to go to work*[32], contracting a deadly disease *through no fault of their own*[33]. There were women affected *many of whom probably did nothing more than wash their husbands’ asbestos coated overalls*[34].

Articles referred to deserving patients … *one of the greatest injustices in Australian history that people who smoked voluntarily can get this drug on the PBS* [subsidised for some lung cancers] *but people with mesothelioma can’t*[35]. A sense of entitlement was reflected in a comparison with other subsidised medicines *why won’t the Government bankroll an effective cancer-fighting drug yet it spends millions of dollars a year providing methadone to more than 30,000 heroin addicts*, [36]*I paid tax all my working life*…*,*[32] and *it is not too much to ask that a Government …shows some commonsense compassion instead of callous, cold-blooded rationalism*[37]. The Asbestos Disease Foundation noted it was *appalling that we can spend so much money going to war and all sorts of other things but when it comes to the medicine and treatment of these tax-paying Australian people we just can find the sense and logic to provide the medicine as required*[38].

When politicians were challenged about the situation, responses referred to *an independent, expert committee* [PBAC]*,*[39] and noting the need for companies to *respect the processes for drug approvals for PBS listing*[39].

There was active lobbying of PBAC and parliamentarians. The then Health Minister dismissed the delivery of a 17,000 signature petition from Bernie Banton as a publicity stunt, earning the ire of the media, and provoking the response that it was *an unacceptable attack on a great Australian*[40]. It resulted in prolonged media coverage around listing of the drug.

The positive decision to list on the PBS was reported as *Bernie wins battle*[41]. We found only one article that provided any detail on the PBAC decision: [the manufacturer] *dropped the price* [by 10%], *changed the way it was packaged to reduce waste and cost and provided more data about how it improved patients’ quality of life*[42].

Bernie Banton commented on possible political interference in the final decision: *Tony Abbott* [Health Minister] *assured me there was no political interference in this decision but I would not have liked to have been him if the decision had gone the other way*[43].

The reporting of the successful listing of pemetrexed was overshadowed by the death of Bernie Banton with tributes and the offer of a State funeral.

### Case study – atomoxetine and methylphenidate for ADHD

In contrast to the apparent support for listing of infliximab and pemetrexed in some newspaper reports, the discourse for the ADHD medicines often related to the dangers of the drug: *deadly side to ADHD treatment,*[44]*when the costs of a well behaved child could be hallucinations or death*[45]. Sometimes, the language of drugs of addiction and war was applied: *Kids’ $10M ADHD habit*; [46]*kiddy-cocaine*; [47,48]*… a further 58 four-year-olds and 13 three-year-olds are also wandering the state like space cadets*; [47]*Australia’s dependency on ADHD drugs*; [46] and *New front opens on ADHD war*[49].

There was also commentary implying the apparently reckless use of these drugs in very young children when perhaps better parenting and discipline were more appropriate: *giving quick and careless prescribers the opportunity to prescribe a drug*, [50]*Prescribe discipline not drugs for children*, [51]*quick fix drugs to solve behavioural problems*, [45]*Has a normal childhood become a disorder?*[52]. There was particular ire at their use in pre-school age children: *Drugs for Toddlers? You must be kidding*, [47] … *you just don’t mess with the heads of babies*, [47] and using *mind-altering drugs… while still in nappies.*[47]

The debate was fuelled by comments from politicians and members of the judiciary expressing their concerns about use of these drugs [53] that *had created a generation of Ritalin kids who were committing violent crimes and coming before the courts*[48]. It was noted that the Prime Minister was…*very worried about the rate of prescribing of ADHD drugs*; [54]*….left open the possibility of a national enquiry into the ADHD epidemic*[48].

In the view of one medical specialist …*I think the numbers* [of children prescribed the drugs] *are not bad actually… we’re not doing such a bad job*[47] (the figures follow the State Government’s ADHD review which found there was no overprescribing of drugs). So difficult was the environment that a specialist involved in developing guidelines for the use of these drugs described the task as a “*contentious project*”, noting “…*there’s a high amount of emotional content from the media feeding into the politicians and presumably other dark forces are working in the background and the whole process has been put on hold*” [55].

Readers of some newspapers were invited to contribute their opinions on a complex social issue in simple yes/no polls: *Does the issue concern you?*[56]*Should taxpayers be forced to subsidise ADHD drugs?*[54]*Should there be a Ritalin age limit?*[57].

## Discussion and conclusion

Most of the medicines considered by the PBAC in 2006–2007 generated little or no newspaper coverage. Medicines that were the focus of media reports included biological agents (monoclonal antibody therapies) and treatments for high profile diseases such as breast cancer, prostate cancer, ADHD, leukaemia and multiple sclerosis. Mentions of government subsidy and cost to the patient were more frequent for high-cost medicines (e.g. pemetrexed). We found no evidence of increased intensity of newspaper reporting close to the time of PBAC meetings.

While overall, most newspaper stories were not published in close proximity to the PBAC consideration for listing there was heightened media activity for a number of specific drugs. Whether the newspapers are actively mounting and promoting campaigns or are the agency for the campaigns of other interest groups is less clear. For both infliximab and pemetrexed, there were well-organised patient groups and disease associations, with petitions delivered to Government. In addition to reporting, at least one media outlet suggested a public campaign as the only chance; [27] and the subsequent successful listing was attributed in part to listening to the public view. In some cases the advocacy is clearer with one newspaper (Sunday Herald Sun) congratulating itself on the success of its media campaign to have treatment with trastuzumab subsidised by the Government; [58] Breast Cancer Network Australia was confident their pressure had “*won the day”*[59].

In a study of the representation of prescription medicines in UK newspapers, Prosser concluded that the media often constructs a discrete, contradictory, and frequently oversimplified set of characterizations about medicines - the contrast of marvellous medicines and dangerous drugs [60]. Our selected case studies illustrate the potential for this, with super effective, wonder drugs like pemetrexed, infliximab, trastuzumab (marvellous medicines) and the potential dangers of medicines to treat ADHD. In the case of ADHD, even the diagnostic criteria are contentious, [61] and the use of the terminology of drugs of addiction and the language of battle [60] (*ADHD war)* will tend to reinforce negative public opinion. Health care professionals seeking to mount a public campaign against perceived over-prescription of these medicines will have also contributed to the negative reporting of these medicines [62]. The effect of the media spotlight and the emotional content of the discourse was sufficient to interfere with the work of clinical experts trying to develop guidelines for use of these drugs.

While the costs of medicines to both patients and the Government featured in many of the stories, there were few challenges to pharmaceutical companies to justify the prices being asked for some of these medicines. There were few references to the decision-making processes used by the PBAC, with its emphasis on evidence of benefit and formal assessment of cost-effectiveness [10]. For pemetrexed, there was little discussion of the potential cost implications of the expected growth in patient numbers of 600 per year to 18,000 cases by 2020 [41] or the projected gain in life-expectancy (proven to offer two or three extra months of life) [63]. Interestingly, there were references to the comparatively cheaper ADHD medicines as costly tablets, [55] generating a bill of almost $10 million per year that taxpayers were forced to pay [46].

In the newspaper reports included in this study, there was little recognition that the formal and independent processes for the listing of drugs on the PBS had served the Australian taxpayer well. The system is acclaimed internationally and is generally believed to provide a consistent and defensible framework for decision-making delivering prices for new medicines lower than most other countries. Among the media reports about failure to fund the HPV vaccine and the pleas to the Health Minister to circumvent the PBAC’s advice in relation to the HPV vaccine, [64] few reports acknowledged that vaccine listing on the NIP was achieved with a substantial reduction in price from the manufacturer [65]. An offer of a price reduction, repackaging to reduce waste and more clinical data led to the final approval of pemetrexed [42]. Intensive lobbying lead to the 2001 decision to fund trastuzumab in a program separate to the PBS; this mechanism to provide medicine access has subsequently been described as “*a mistake best not repeated*” [59]. Applying different decision-making criteria for specific diseases creates difficult precedents for both government and the PBAC.

The case studies for infliximab and pemetrexed illustrate the use of identifiable victims to put a human face on the disease and its consequences. Apart from engaging reader attention, it also invokes the “rule of rescue”, i.e. the imperative people feel to rescue identifiable individuals facing avoidable death, [66] and can increase the likelihood of achieving the objective of gaining access to expensive medicines [14].

Our study was limited to newspaper reports of medicines drawn from a single news database, ANZ Proquest Newsstand. While the repository does not include all newspapers available in Australia and does not include online and television news stories, the scope and size of the readership of the included newspapers means that the discourse observed in these articles is likely to be reflective of the information and views offered to the Australian public. There was often syndication of stories to other news outlets, or use in more than one edition of a newspaper; we counted these as separate stories as an editorial decision had been made to reproduce the story and the readership was likely to be different. There were few useful classification frameworks available to guide our analysis of article content; our experience was that a numerical count of drug mentions was not always a good guide to article focus and content.

The results of this study suggest that mostly the PBAC can apply its rigorous evidence-based assessments away from the public gaze, however there are some decisions that have to be taken in a politically and emotionally charged environment. The media have a legitimate right to report and to give voice to the range of viewpoints. Some diseases lend themselves to more sympathetic attention than others (breast cancer and mesothelioma compared to bowel cancer; [67,68]) however media prominence is a poor basis for decision-making. These funding decisions will become increasingly difficult as the manufacturers of new and expensive medicines seek public subsidy of agents that offer sometimes small clinical gains over existing therapies. In this environment, the media can play an important role in improving public understanding of the issues around resource allocation. Guidelines from professional associations such as the Australian Press Council, [69] use of checklists for journalists on key elements to include in their stories, [70,71] and dedicated specialist health journalists [71] may all help in improving the quality of health reporting.

## Appendix I

List of all drugs included in the study by number of articles referring to the medicine:

**≥50 articles:** HPV vaccine, Trastuzumab, Pemetrexed, Botulinum toxin, Lapatinib, Methylphenidate, Atomoxetine, Tdap vaccine (Adacel).

**≥20 to 49 articles**: Infliximab, Rotavirus vaccine, Varenicline, Docetaxel, Sildenafil, Paclitaxel, Imatinib, Ranibizumab, Fentanyl, Natalizumab

**≥10 to 19 articles:** Imiquimod, Rituximab, Rosuvastatin, Insulin glargine, Adalimumab, Levonorgestrel, Topiramate, Risperidone, Cetuximab, Ezetimibe, Ezetimibe with Simvastatin, Budesonide with Eformeterol, Cinacalcet, Fluticasone with Salmeterol, Pioglitazone, Etanercept, Insulin detemir, Letrozole

**1 to 9 articles:** Exemestane, Pimecrolimus, Risedronate, Bortezomib, Quetiapine, Famciclovir, Zonisamide, Calcipotriol, Paliperidone, Sevelamer, Ziprasidone, Anecortave, Darunavir, Pegfilgrastim, Trandolapril with Verapamil, Abatacept, Alendronate, Bicalutamide with Goserelin, Dasatinib, Entecavir, Ibandronic acid, Macrogol, MMRV vaccine, Rosiglitazone with Metformin, Strontium, Triptorelin

**No articles:** Alendronate with Vitamin D3, Amlodipine with Atorvastatin, Aprepitant, Deferasirox, Epoprostenol, Leflunomide, Moxonidine, Olmesartan with Hydrochlorothiazide, Posaconazole, Ramipril with Felodipine, Risedronate with Calcium, Sitaxentan, Tacrolimus, Thyrotropin alfa-rch, Tipranavir, Travoprost with Timolol, Vinorelbine

## Abbreviations

ADFA: Asbestos diseases foundation of Australia; ADHD: Attention deficit hyperactivity disorder; HPV: Human papillomavirus; NIP: National Immunisation Program; PBAC: Pharmaceutical Benefits Advisory Committee; PBS: Pharmaceutical Benefits Scheme; Tdap: Tetanus diphtheria, acellular pertussis

## Competing interests

The authors declare that they have no competing interests. DN was appointed as a member of the PBAC in 2012, but had no role in PBAC decision-making when this work was undertaken. Ethics Committee approval was not required for this study as all material analysed are in the public domain. There was no external funding for this study.

## Authors’ contributions

JR conceived of the study, and participated in its design and coordination, was involved in interpreting the data, and drafted the manuscript. EW was involved in collection, interpretation and analysis of the data, and helped draft the manuscript. MB was involved in collection, interpretation and analysis of the data, and helped draft the manuscript. DN conceived of the study, and participated in its design and coordination, and helped draft the manuscript. The authors had full access to all data in the study. All authors read and approved the final manuscript.

## Pre-publication history

The pre-publication history for this paper can be accessed here:

http://www.biomedcentral.com/1471-2458/13/489/prepub
